# The spiked helmet sign in a patient with erysipelas: an alarming electrocardiogram sign (a case report)

**DOI:** 10.11604/pamj.2023.46.58.40438

**Published:** 2023-10-17

**Authors:** Salihou Fall, Sameh Ben Farhat, Ahmed Chelly, Hella Kaddour, Saeb Ben Saad, Ahmed Mohamed El Hedi, Mehdi Slim, Houssem Thabet, Sami Ouannes, Rym Gribaa, Aymen Elhraiech, Neffati Elyes

**Affiliations:** 1 Cardiology Department, Sahloul University Hospital, Faculty of Medicine Ibn El Jazzar of Sousse, University of Sousse, Sousse, Tunisia,; 2 Emergency Medical Service Department (SAMU03), Sahloul University Hospital, Faculty of Medicine Ibn El Jazzar of Sousse, University of Souse, Sousse, Tunisia

**Keywords:** Electrocardiogram, spiked helmet sign, STEMI mimic, prognosis, case report

## Abstract

Early diagnosis of the spiked helmet sign is challenging. This ST-elevation myocardial infarction mimic was first described in 2011 by Littmann and colleagues and was linked to severe non-coronary pathologies, with a high risk of mortality. We present a case of a 60-year-old female patient who developed severe erysipelas with sepsis associated with severe hypokalemia. She had a spiked helmet sign on her routine electrocardiogram at hospital admission. We performed a coronary angiogram that showed no culprit artery. She developed afterward an ischemic stroke. Through intensive management of the patient’s sepsis and electrolyte disturbance, she had a favorable outcome.

## Introduction

Early detection of an ST-segment Elevation Myocardial Infarction (STEMI) is crucial, but there are rare cases where STEMI mimics can mislead to its diagnosis [1]. The “spiked helmet sign” is an electrocardiogram marker associated with severe non-coronary causes [2]. Littmann and colleagues first described this sign in 2011 [2]. It manifested as a dome-shaped ST-segment elevation, where the upward shift occurs before the onset of the QRS complex [2]. Reported cases observed this sign of intrathoracic diseases such as pneumothorax, intrabdominal surgical pathologies such as bowel perforations [3], subarachnoid hemorrhage, sepsis, and severe metabolic disturbance [4]. A delay in early and prompt management of these underlying causes can result in fatal outcomes. The spiked helmet sign was high-risk mortality in the case series (around 75%) [2]. Despite its recognition, the exact underlying pathophysiology remains unclear [4]. We report an original case of a 60-year-old patient admitted for erysipelas. During hospitalization, her electrocardiogram showed a spiked helmet sign.

## Patient and observation

**Patient information:** a 60-year-old female with a previous history of hypertension, who had discontinued her treatment, was initially admitted to a rural hospital's medical department to treat erysipelas in her right leg. The patient's medical history did not include any reports of chest pain.

**Clinical findings:** on physical examination, the patient had a Glasgow coma scale of 15, a temperature measuring 39.2°C, and her cardiorespiratory assessment revealed normal findings. She had an erysipelas in her right leg without any necrotic lesions.

**Figure 1:**
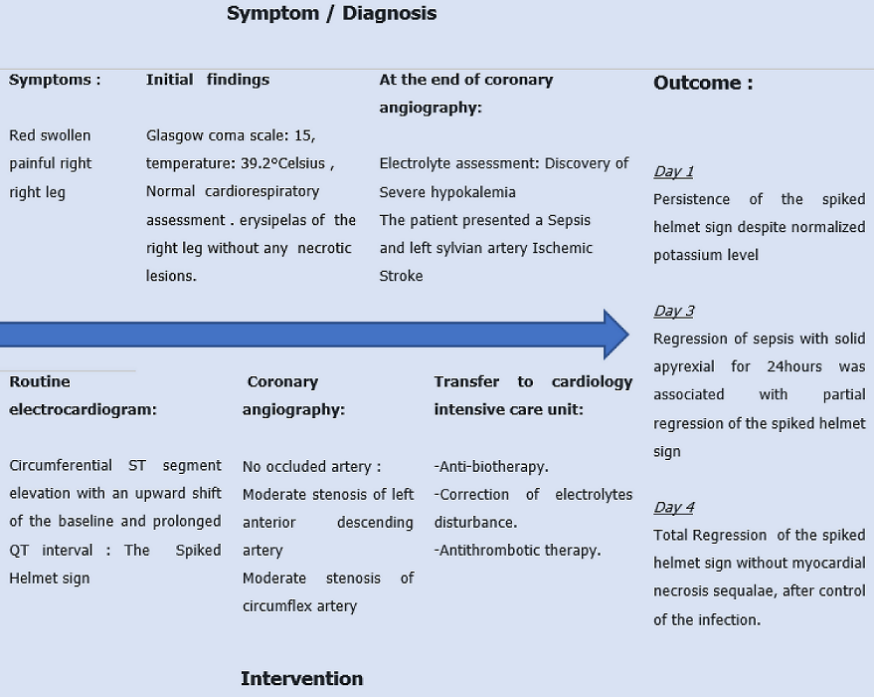
timeline of the current episode from admission to management

**Timeline of the current episode:** the main steps from the patient´s admission to management are summarized in (Figure 1).

**Figure 2:**
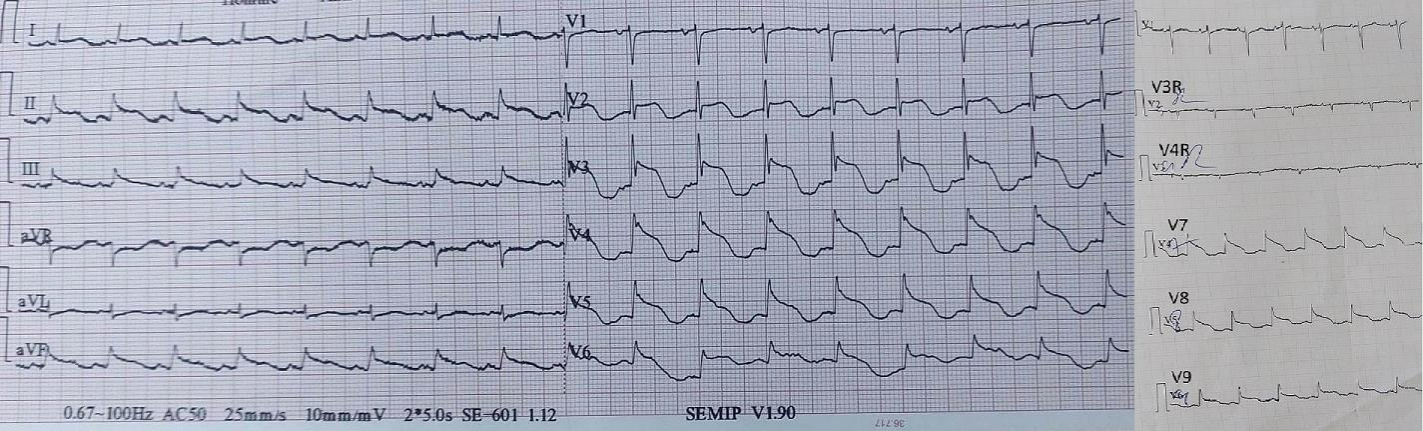
patient’s 18-leads electrocardiogram: a regular sinus rhythm with an upward shift of the TP segment, with thin QRS complex and a dome-shaped ST-segment elevation in leads: DI DII DIII AVF V2 V3 V4 V5 V6 V7 V8 V9, and ST segment depression in AVR and V1; with a prolonged QT interval corrected QT (Bazett) at 516 milliseconds

**Figure 3:**
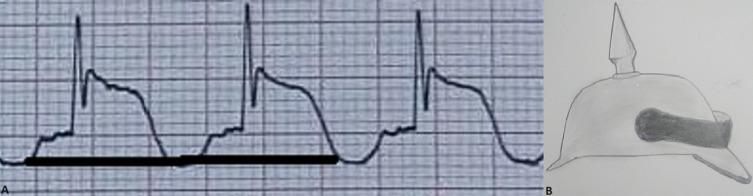
enlargement of the electrocardiogram showing the resemblance of the electrocardiogram pattern to the German spiked helmet from the 19^th^ century known as “Picklehauben”

**Figure 4:**
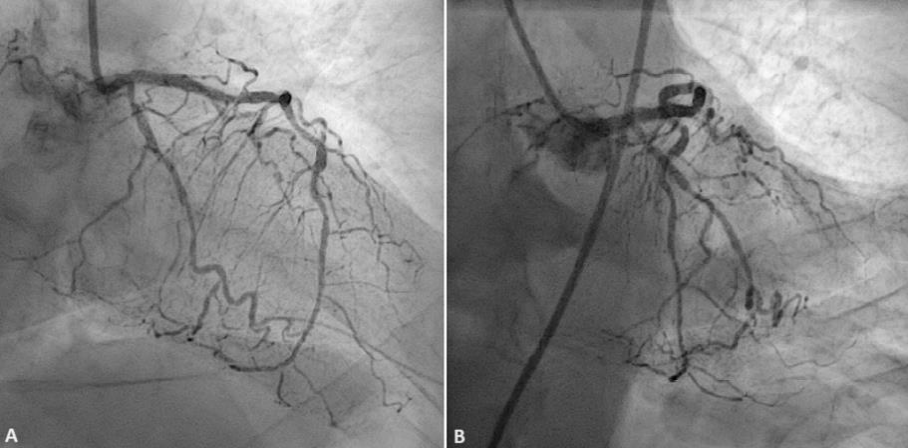
patient’s coronary angiogram: normal-sized left main coronary artery with mild calcification and no significant lesion; the left anterior descending coronary artery presented multiple staged intermediate lesions and significant stenosis involving the ostial and proximal segments of the circumflex artery

**Diagnostic assessment:** upon admission, a routine electrocardiogram revealed a diffuse ST-segment elevation and a prolonged QT interval [corrected QT (Bazett) = 516 milliseconds] (Figure 2 and Figure 3). Initially, we diagnosed a circumferential STEMI leading to the administration of a loading dose of antithrombotic therapy. The patient was subsequently transferred to our cardiac catheterization room for immediate coronary angiography and potential primary percutaneous coronary intervention. The invasive exploration showed a normal-sized left main coronary artery with mild calcification and no significant lesion. The left anterior descending coronary artery showed multiple staged intermediate lesions. A significant stenosis involved the ostial and proximal circumflex artery (Figure 4). However, we were unable to visualize the right coronary artery. Following coronary angiography, she presented a predominantly brachio-facial right hemiparesis, with ipsilateral central facial paralysis, associated with Broca's aphasia, and swallowing disorders. Cerebral and supra-aortic trunk computed tomography scans showed normal results, confirming a diagnosis of left superficial sylvian ischemic stroke. The patient was transferred to our cardiovascular intensive care unit and she remained clinically stable. The follow-up electrocardiograms showed the same aspect. A transthoracic echocardiogram performed did not reveal any abnormal findings. Initial blood tests revealed hypokalemia: 2.3 mmol/l, hypocalcemia: 2.02 mmol/l, active inflammatory signs C-reactive protein: 373 mg/L and leukocytosis, white blood cells: 20,900cells/mm^3^. Additionally, the complete blood count showed hypochromic microcytic anemia: 11.3 g/dl, with mean corpuscular volume: 76 femtoliters (fl) post-coronary angiography. The ultrasensitive troponin level was at 7700ng/l. Creatine-phosphokinase at 4400 IU/l, lactate dehydrogenase at 484 UI/l, and liver transaminases Aspartate Transaminase, and Alanine Transaminase respectively at 239 and 56 IU/l.

**Diagnosis:** the patient presented a STEMI mimic, the “Spiked helmet sign” concomitant with a right leg erysipelas, sepsis, and severe hypokalemia. This was followed by the occurrence of a left superficial sylvian ischemic stroke.

**Therapeutic interventions:** we initiated an anti-ischemic treatment in conjunction with appropriate antibiotic therapy and correction of electrolyte disorders.

**Follow-up and outcome of interventions:** within three days of initiating antibiotic therapy the patient became apyrexial and presented a regression of local signs of erysipelas. During the course of treatment, the ST-segment elevation persisted for 48 hours, followed by a gradual regression. After four days, we observed a complete resolution, and there were no lasting sequelae of myocardial necrosis.

**Informed consent:** we could not reach the patient after her discharge to obtain her consent. However, we did not describe any information enabling her identification, ensuring the protection of the patient´s privacy.

## Discussion

The spiked helmet sign is an electrocardiogram pattern, STEMI mimics that Littmann and colleagues first described in 2011 in a series of 8 patients with severe extracardiac pathologies; 6 of whom died within 10 days of the spiked helmet sign diagnosis [2]. Therefore, it has been hypothesized as a marker of high-risk mortality [2]. The spiked helmet sign is characterized by a dome-shaped ST-segment elevation preceded by an upward shift of the baseline preceding the onset of the QRS complex [2], and is often associated with a prolonged QT interval and invisible p waves [5] (Figure 3). While This electrocardiogram pattern was first described as occurring in the inferior leads DII, DIII, and AVF [2], however, other cases reported antero-septal and lateral lead presentations [6]. The spiked helmet sign was associated with a variety of severe pathologies including sepsis, gastro-intestinal perforation, hypoxemic pneumonia, viral myocarditis [6], subarachnoid hemorrhage, and anoxic-ischemic axonal lesions [5]. Cases of intrathoracic hyperpressure in patients on invasive ventilation or with pneumothorax [5] are also reported, as well as concomitant severe ionic disorders such as hypomagnesemia [7]. This sign was also described in a patient with Takotsubo syndrome [8]. Notably coronary angiography in these patients often showed normal coronary arteries and markers of myocardial necrosis were negative.

The underlying pathophysiology of the spiked helmet sign remains unknown. However, several hypotheses have been proposed. One suggests that this electrocardiogram pattern was an artifact due to synchronization between the diaphragmatic contractions and the ventricular systoles. This phenomenon has been observed through spirography [9]. This synchronization was due to muscular hyperexcitability secondary to ionic disorders, and to direct mechanical stimulation of the diaphragm by the lower wall of the heart or by the phrenic nerve [2]. Another hypothesis was based on the QT interval prolongation associated with very wide negative t waves. It suggests an adrenergic hyperstimulation in response to stressful situations like sepsis and subarachnoid hemorrhage [10]. Lastly, some authors have proposed that it could be an electrocardiogram artifact secondary to epidermal distension, as seen in cases of intrathoracic or intra-abdominal hypertension [2].

On one hand, several explanations can be considered in our patient for the spiked helmet sign, firstly the presence of hypokalemia could support the hypothesis of QT interval prolongation, however, the “spiked helmet sign” persisted after the correction of the patient´s electrolyte disorders. Suggesting that hypokalemia alone cannot fully explain this electrocardiogram pattern. Secondly, the associated state of severe sepsis could support the hypothesis of adrenergic hyperstimulation, particularly given that the spiked helmet sign regressed after the control of the infection. On the other hand, several arguments did not support the ischemic origin of ST-segment elevation. Notably, the coronary lesions observed on angiography were not consistent with the extent of cardiac repolarization abnormalities observed on the electrocardiogram. Furthermore, the ST segment elevation persisted for 48 hours and then regressed without electrical sequelae of myocardial necrosis. Finally, there were no concomitant echocardiographic wall motion abnormalities on transthoracic echocardiography.

First, the originality of our case consists in the diffuse character of the “spiked helmet sign” in almost all electrocardiogram leads, then despite the poor prognosis associated with this electrocardiogram entity the patient´s outcome was favorable.

## Conclusion

The value of the “spiked helmet sign” as a marker of high-risk mortality has yet to be established. A precise interpretation of the electrocardiogram particularly the baseline, is essential to accurately identify this STEMI mimic. The early recognition of the pattern helped in the early and intensive management of the patient´s sepsis and electrolyte disturbance and led to a favorable outcome.
